# Application and prospect of targeting innate immune sensors in the treatment of autoimmune diseases

**DOI:** 10.1186/s13578-022-00810-w

**Published:** 2022-05-26

**Authors:** Jun Liu, Hui Zhang, Yanhong Su, Baojun Zhang

**Affiliations:** 1 Department of Pathogenic Microbiology and Immunology, School of Basic Medical Sciences, Xi’an Jiaotong University, Xi’an, 710061 Shaanxi China; 2 Institute of Infection and Immunity, Translational Medicine Institute, Xi’an Jiaotong University Health Science Center, Xi’an, 710061 Shaanxi China; 3 NHC Key Laboratory of AIDS Immunology (China Medical University), National Clinical Research Center for Laboratory Medicine, The First Affiliated Hospital of China Medical University, Shenyang, 110001 China; 4 Key Laboratory of Environment and Genes Related to Diseases, Xi’an Jiaotong University, Xi’an, 710061 Shaanxi China; 5 Basic and Translational Research Laboratory of Immune Related Diseases, Xi’an, 710061 Shaanxi China

**Keywords:** PRR, Autoimmune disease, Inflammation, Innate signaling, Immunotherapy

## Abstract

Dysregulation of auto-reactive T cells and autoantibody-producing B cells and excessive inflammation are responsible for the occurrence and development of autoimmune diseases. The suppression of autoreactive T cell activation and autoantibody production, as well as inhibition of inflammatory cytokine production have been utilized to ameliorate autoimmune disease symptoms. However, the existing treatment strategies are not sufficient to cure autoimmune diseases since patients can quickly suffer a relapse following the end of treatments. Pattern recognition receptors (PRRs), including Toll-like receptors (TLRs), Nod-like receptors (NLRs), RIG-I like receptors (RLRs), C-type lectin receptors (CLRs) and various nucleic acid sensors, are expressed in both innate and adaptive immune cells and are involved in the development of autoimmune diseases. Here, we have summarized advances of PRRs signaling pathways, association between PRRs and autoimmune diseases, application of inhibitors targeting PRRs and the corresponding signaling molecules relevant to strategies targeting autoimmune diseases. This review emphasizes the roles of different PRRs in activating both innate and adaptive immunity, which can coordinate to trigger autoimmune responses. The review may also prompt the formulation of novel ideas for developing therapeutic strategies against autoimmune diseases by targeting PRRs-related signals.

## Background

Autoimmune diseases are generally considered uncommon, but they significantly affect the mortality and morbidity of the population. Rigorous epidemiological studies have reported that approximately 3 ~ 5% of the population suffers from autoimmune diseases [1]. In principle, the occurrence of autoimmune disease is triggered by specific recognition of self-antigens by adaptive immune cells. In this context, T cells, which recognize autopeptides presented by antigen-presenting cells (APCs), become autoreactive effector cells capable of producing inflammatory cytokines. Activated T cells also help B cells produce antibodies against self-tissues. Excessive inflammation and autoantibodies finally lead to different types of pathogenic symptoms. Currently, several classes of drugs, for example, non-steroidal anti-inflammatory drugs, glucocorticoids, immunosuppressive drugs (methotrexate, leflunomide and cyclophosphamide) and biological drugs (TNF inhibitors, IL-1 inhibitors, anti-T-cell co-stimulation fusion protein and anti-B-cell monoclonal antibody, et al.), are applied in clinic for autoimmune disease treatment. However, some treatments show limited efficacy and serious long-term side effects [2–11]. Moreover, the diseases frequently relapse in patients upon treatment removal [12–18]. Therefore, it is necessary to explore the complex mechanisms of autoimmunity and optimize treatment strategies.

Innate immunity is the first line to defend against variant pathogens by recognizing pathogen-associated molecular patterns (PAMPs) and danger-associated molecular patterns (DAMPs) by pattern recognition receptors (PRRs). PAMPs, such as polysaccharides, glycolipids, lipoproteins, nucleotides and nucleic acids, are commonly expressed by various pathogens, while DAMPs are endogenous host molecules released from stressed or dying cells [19, 20]. PRRs, including Toll-like receptors (TLRs), Nod-like receptors (NLRs), RIG-I like receptors (RLRs), C type lectin receptors (CLRs) and various nucleic acid sensors, recognize PAMPs and DAMPs and become activated to initiate innate immune responses to produce proinflammatory molecules and clear various pathogens. However, overactivation of PRRs and excessive inflammation are involved in various autoimmune diseases, such as psoriasis, systemic lupus erythematosus (SLE), and rheumatoid arthritis (RA) [21–24]. In addition, PRRs are also expressed in adaptive immune cells and trigger autoimmune responses directly by shaping adaptive immunity to a proinflammatory phenotype [25–29]. Therefore, targeting PRRs may be a promising strategy for treating autoimmune diseases. Indeed, a number of small molecules, peptides, antibodies, proteins, nanoparticles and drugs targeting PRRs have been investigated. Herein, we summarized the signaling pathways of PRRs, the potential roles of PRRs in activating autoimmunity, and their applications in treating autoimmune diseases.

## TLRs and autoimmune diseases

### TLRs and their ligands

TLRs, as type I transmembrane proteins, are composed of an extracellular domain containing tandem copies of leucine-rich repeats (LRR) responsible for ligand recognition, a transmembrane domain, and an intracellular domain, also named the Toll interleukin-1 receptor homology (TIR) domain, responsible for signal transduction. TLRs can recognize 10 different PAMP types in humans and are expressed in immune cells, including dendritic cells (DCs), macrophages, natural killer (NK) cells, T and B lymphocytes and nonimmune cells such as epithelial cells, endothelial cells and fibroblasts [30]. TLR1, 2, 4, 5, 6 and 10 are mainly localized on the cell surface and are involved in the recognition of proteins and lipopeptides. Additionally, TLR3, 7, 8 and 9 are mainly localized in endosomes and are involved in the detection of nucleic acids [26, 31–34]. More specifically, TLR2 forms heterodimerizations with either TLR1 or TLR6, which respond to triacylated lipopeptides from gram-negative bacteria or diacylated lipopeptides from mycoplasma and gram-positive bacteria, respectively [34, 35]. TLR4 recognizes lipopolysaccharide (LPS) principally from the germ-negative bacterial cell wall. TLR5 recognizes flagellin, the major structural protein of bacterial flagella. In addition, TLR3 recognizes double-stranded RNA (dsRNA), TLR7 and TLR8 recognize single-stranded RNA (ssRNA), and TLR9 recognizes unmethylated CpG DNA from viruses, bacteria, and the host [34]. TLR10 is mostly expressed on the plasma membrane of monocytes. Thus far, the ligand of TLR10 remains to be illuminated. Recently, TLR10 was reported to play an anti-inflammatory role by suppressing the production of IL-6, TNF-α and IL-1β and inhibiting the activation of T cells by DCs [36, 37].

### The downstream pathways of TLRs signaling

**Fig. 1:**
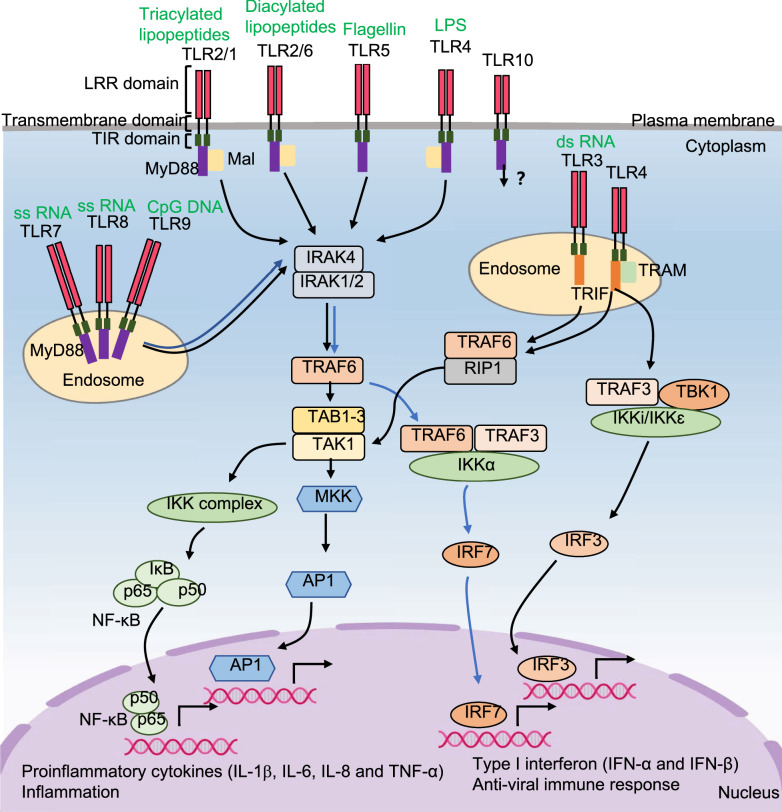
The signaling pathways for TLRs. Cell membrane receptors, TLR2/1, TLR2/6, TLR5 and TLR4, and endosomal membrane receptors, TLR7, TLR8 and TLR9 are activated by their ligands. Then they interact with MyD88 and recruit the IRAK complex and TRAF6 to activate TAK1. TAK1 not only activates IKK complex to induce NF-κB activation, but also activates MKK to induce AP1 activation, which result in the transcription of proinflammatory cytokines (IL-1β, IL-6, IL-8 and TNF-α). Activation of TLR7, TlR8 and TLR9 trigger the IRAK-TRAF6-TRAF3-IKKα-dependent activation of IRF7, leading to transcription of type I IFNs. TLR3 signals not only through TRIF-TRAF6-RIP1-TAK1-dependent activation of NF-κB and AP1 pathways, but also through TBK1 dependent activation of IRF3 pathway. TLR4 initially signals on cell membrane through MyD88-dependent pathway. Subsequently, receptor internalization into endosomes triggers TRIF-dependent pathway, but additionally requires TRAM. The ligand and signaling pathway of TLR10 remain unclear

Four major adaptor proteins, myeloid differentiation primary response 88 (MyD88), MyD88 adapter like (Mal), TIR-domain containing adapter inducing interferon-β (TRIF) and TRIF-related adaptor molecule (TRAM), and two pathways, the MyD88-dependent pathway and TRIF-dependent pathway, are involved in TLR signaling (Fig. 1). Most TLRs use the MyD88-dependent pathway, except for TLR3. TLR2/1, TLR2/6 and TLR4 use MyD88 to initiate signaling in the presence of Mal, while TLRs 5, 7, 8 and 9 do not need Mal. MyD88 interacts with interleukin 1 receptor associated kinase 4 (IRAK-4), and IRAK-4 then phosphorylates IRAK-1 and IRAK-2, which in turn activate TNF receptor associated factor 6 (TRAF6). TRAF6 then activates TGF-β-activated kinase 1 (TAK1) in cooperation with TAK1-binding proteins (TAB1-3). TAK1 activates NF-κB and AP1 through the IKK and MAPK pathways, which induce the transcription of proinflammatory cytokines. In addition, TLR7, 8, and 9 trigger the IRAK-TRAF6-TRAF3-IKKα-dependent activation of IRF7, leading to transcription of type I interferons. Moreover, TLR3 signals through a TRIF-dependent pathway. TLR3 not only induces proinflammatory cytokines through TRAF6-receptor-interacting protein-1 (RIP-1)-TAK1-dependent activation of NF-κB and AP1 but also induces type I interferons by triggering TANK-binding kinase 1 (TBK1)-dependent activation of IRF3 [38–43]. After binding to LPS at the cell surface, TLR4 initially signals through the MyD88 pathway. Subsequently, receptor internalization into endosomes triggers the TRIF-dependent pathway but additionally requires TRAM [38, 44, 45].

### Relationship between TLRs and autoimmune diseases

TLRs are essential for innate immunity against infection. However, inappropriate TLR responses cause acute and chronic inflammation and further autoimmune diseases [46, 47]. For example, TLR2/1 signaling activation induces proinflammatory cytokines, such as IL-1β, TNF and IL-6, and is implicated in RA [48, 49]. TLR7 and TLR9 signaling activation-induced type I interferons are implicated in SLE [47, 50]. Moreover, some TLRs activate DCs and macrophages to produce proinflammatory cytokines and chemokines and upregulate costimulatory molecules, which in turn regulate adaptive immune cells and lead to autoimmune diseases [44, 51]. For example, TLR2 and TLR4 activation induces inflammation in the liver. The sustained increase in IL-6 and IL-12 inhibited the suppressive function of Tregs. Meanwhile, increasing IL-12 and IL-4/IL-25 promoted Th1 and Th2 responses, respectively. Thus, immune tolerance against liver tissue is disrupted, which results in autoimmune hepatitis (AIH) [52]. TLR2- or TLR4-mediated stimulation was also reported to promote DCs to produce IL-10, induce an augmented Th2 immune response, and aggravate systemic sclerosis (SSc) [53]. In addition, TLR7-mediated IL-6, IL-1β, and IL-23 production by DCs promotes the Th17 response, which results in severe experimental autoimmune uveitis (EAU) [54]. Hence, TLRs expressed on innate immune cells may promote autoimmune diseases by boosting inflammation and impacting adaptive immune responses.

In addition, some adaptive immune cells can express TLRs, which are involved in autoimmune disease [55, 56]. For example, TLR2 activation in CD4^+^ T cells is involved in experimental autoimmune encephalomyelitis (EAE) development by augmenting the Th17 response [27], while TLR4 activation induces EAE development by augmenting both Th1 and Th17 responses [57]. Cleonice et al. reported that the expression of TLR2, TLR4 and TLR9 was significantly higher on CD4^+^ and CD8^+^ T lymphocytes from multiple sclerosis (MS) patients than on those from healthy individuals. The expansion of different Th17 phenotypes expressing TLR2, TLR4 and TLR9 was associated with MS disease activity [58]. Furthermore, some TLRs can regulate the functions of Tregs. The activation of TLR2, TLR8 and TLR9 by their ligands can abrogate the suppressive function of Tregs [59, 60]. In contrast, the TLR5 ligand flagellin can enhance the suppressive function of Tregs by enhancing FOXP3 expression [61]. The TLR3 ligand polyI:C was reported to induce IFN-γ^+^Foxp3^+^ Tregs that have potent immunosuppressive functions and prevent food allergy (FA) development in mice [59]. Moreover, B cells were reported to recognize nucleic acids via TLR7 and TLR9 and produce autoreactive antibodies, promoting the development of SLE [62–64]. Thus, TLRs expressed on adaptive immune cells may promote autoimmune diseases, although there is a debate on Treg functions regulated by different TLRs.

## NLRs and autoimmune diseases

### NLRs and their ligands

NLRs are distributed in the cytoplasm and mostly recognize invading bacteria. A total of 22 NLRs are detected in humans. All NLRs contain a C-terminal LRR, a central nucleotide-binding oligomerization domain (NOD or NACHT), and an N-terminal effector domain. The N-terminal effector domain binds with adaptor molecules and triggers downstream signals. NLRs can be divided into five subfamilies according to unique N-terminal effector domain: NLRA containing an acidic transactivation domain (AD), NLRB containing a baculovirus inhibitor of apoptosis protein repeat (BIR) domain, NLRC containing a caspase activation and recruitment domain (CARD), NLRP containing a pyrin domain (PYD) and NLRX containing an unidentified domain or mitochondria-localization sequence (MTS) [65–67]. The NLRA subfamily has a single member, MHC-II transcription activator (CIITA), whose primary function is to regulate the expression of MHC-II in different populations of APCs [67]. NLRB, also known as neuronal apoptosis inhibitory protein (NAIP), is expressed in the central nervous system, placental liver, spleen, lung, and peripheral blood leukocytes and exerts antiapoptotic effects [22, 68]. NLRC consists of five members (NLRC1–5). NLRC1 (NOD1) is expressed in a variety of cells, including epithelial cells, stromal cells, and endothelial cells, and recognizes g-D-glutamylmeso-diaminopimelic acid (iE-DAP), which is produced by most gram-negative and several gram-positive bacteria. NLRC2 (NOD2), mainly expressed in monocytes, DCs, macrophages, B cells and T cells, recognizes the muramyl dipeptide peptidoglycan (MDP) motifs present among both gram-positive and gram-negative bacteria and ssRNA of virus [69]. NLRC3 is expressed in myeloid cells, epithelial cells and T cells, and its ligands are largely unknown [70]. NLRC4 is expressed in epithelial cells and functions together with the NLRB protein in sensing bacterial flagellin and components of the bacterial T3SS and in forming inflammasomes [71]. NLRC5 is mostly expressed in bone marrow, lymph nodes, spleen, and mucosal surfaces, such as the lung, small intestine, colon, and uterus. It regulates MHC I transcription and expression and complements CIITA in T cell recognition [72]. The NLRP family consists of several members, of which NLRP1, NLRP3, NLRP6, NLRP7 and NLRP12 are reported to form inflammasomes in response to microbial pathogens, UV light, crystalline particles, potassium efflux and mitochondrial reactive oxygen species (ROS) production [73]. NLRP10 is widely expressed in myeloid cells, epithelial cells, and keratinocytes and plays a role in immunoregulation other than invading pathogen recognition function [74–77]. Until now, NLRX1, the only member of the NLRX family, was reported to recognize viral dsRNA in airway epithelial cells and result in barrier dysfunction in airway epithelia by inducing ROS production [67, 78].

### The downstream pathways of NLRs signaling

**Fig. 2:**
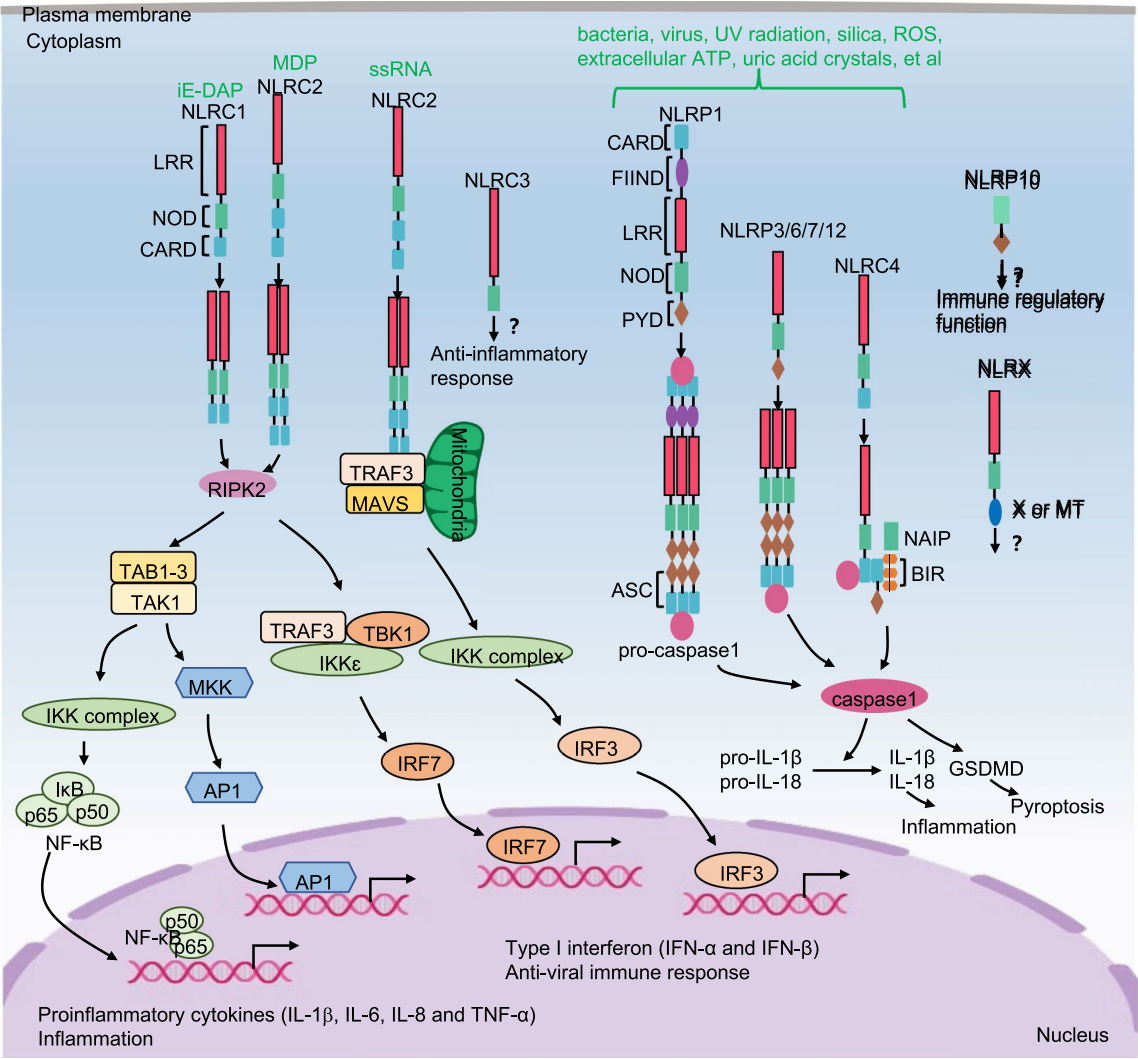
The signaling pathways for NLRs. Activated NLRC1 and NLRC2 respectively self-oligomerize, and then recruit RIPK2. RIPK2 not only activates TAK1 to induce IKK-NF-κB and MAPK-AP1 pathway, but also interacts with TRAF3 to induce TBK1-IKKε complex and further activates IRF7, which result in the transcription of proinflammatory cytokines (IL-1β, IL-6, IL-8 and TNF-α) and type I IFNs, respectively. Once NLRC2 is activated by ssRNA of virus, NLRC2 oligomerizes and then interacts with MAVS protein and TRAF3 to activate IRF3 pathway, which induces expression of type I IFNs. Activated NLRC4/NAIP and NLRP members (NLRP1, 3, 6, 7 and 12) recruit ASCs and caspase-1 to form the basis of the inflammasome. The activated caspase-1 not only cleaves pro-IL-1β and pro-IL-18 into IL-1β and IL-18, but also triggers pyroptosis by cleaving GSDMD. So far, the downstream signaling pathways of NLRC3, NLRP10 and NLRX1 remain to be investigated

NLRC1 and NLRC2 are in an autoinhibited monomeric state in the cytoplasm. Upon ligand recognition, NLRC1 and NLRC2 self-oligomerize and recruit receptor-interacting serine/threonine-protein kinase 2 (RIPK2) through homotypic CARD-CARD interactions. RIPK2 then recruits and activates TAK1, which induces the activation of the IKK-NF-κB and MAPK-AP1 signaling pathways, resulting in the expression of proinflammatory cytokines [79]. RIPK2 also activates the TRAF3-IKKε-IRF7 signaling pathway, resulting in the production of type I IFNs. Once NLRC2 is activated by viral ssRNA, NLRC2 oligomerizes and then interacts with the mitochondrial antiviral signaling (MAVS) protein and TRAF3 to activate IRF3. Afterward, IRF3 translocates into the nucleus and induces type I IFN expression [80]. Activated NLRC4/NAIP and NLRP members (NLRP1, 3, 6, 7 and 12) recruit apoptosis-associated speck-like protein (ASCs) containing a CARD and caspase-1 to form the basis of the inflammasome. Activated caspase-1 cleaves pro-IL-1β and pro-IL-18 into their active forms, IL-1β and IL-18. It also cleaves gasdermin D (GSDMD) and then triggers pyroptosis [65] (Fig. 2). Currently, the signaling mechanisms of NLRC3, NLRP10 and NLRX1 are unclear and need further investigation.

### Relationship between NLRs and autoimmune diseases

NLRs play variable roles in the development and progression of autoimmune diseases by affecting innate immunity. For example, NLRC1 was reported to be overexpressed in ulcerative colitis (UC) patients and caused excessive activation of the innate immune response in a mucosal cell line [81]. Unlike NLRC1, the loss of NLRC2 functions resulted in downregulated pathogen clearance capacity of the host and maintained lasting infection and chronic inflammation, which was associated with the development of Crohn’s disease (CD) [69, 82]. Additionally, NLRC2 polymorphisms were reported to be involved in Blau syndrome [80]. Moreover, an inherited mutation of NLRC4 in familial cold autoinflammatory syndrome (FCAS) was observed. The mutant NLRC4 activated caspase-1 and resulted in the increased secretion of IL-1β [83]. Transgenic mice that expressed mutant NLRC4 under the invariant chain promoter developed dermatitis and arthritis, accompanied by bone erosion [83]. Therefore, NLRC4 is a causative gene for FCAS and plays roles in the pathogenesis of human inflammatory diseases [83].

In addition, NLRs also influence the development of autoimmune diseases by regulating adaptive immune responses. NLRC3 is expressed both in CD4^+^ T cells and in DCs and limits autoimmunity by different pathways [70, 84]. Uchimura et al. revealed that NLRC3 functioned as a negative regulator affecting the proliferation of both Th1 and Th17 cells, limiting IFN-γ and TNF expression by CD4^+^ T cells and restricting autoimmunity by attenuating T cell signaling and metabolic pathways [70]. Moreover, NLRC3-overexpressing DCs reduced EAE progression by attenuating the antigen-presenting function of DCs via the p38 signaling pathway. NLRC3 reduced DCs’ ability to activate and polarize CD4^+^ T cells into Th1 and Th17 subsets [84]. Therefore, NLRC3 serves as an anti-inflammatory mediator to regulate the T cell response, either directly or indirectly. In addition, NLRC1 and NLRC2 were shown to contribute to the induction of mucosal Th1 and Th17 immune responses during infection [80]. Similarly, NLRP10 and NLRP12 have been reported to display anti-inflammatory functions or pro-inflammatory functions in different pathogen infections [74–77, 85–87]. However, their roles in the development of autoimmune diseases by regulating adaptive immune responses remain unknown.

## RLRs and autoimmune diseases

### RLRs and their ligands

RLRs localize in the cytosol in various cell types, such as myeloid cells, epithelial cells, and cells of the central nervous system [88]. RLRs are key sensors of viral infection and belong to the DExD/H (x can be any amino acid residue) box RNA helicase family. This protein family consists of three members: retinoic acid-inducible gene I (RIG-I), melanoma differentiation-associated gene 5 (MDA5), and laboratory of genetics and physiology 2 (LGP2). All RLRs contain a central DEAD box with RNA helicase activity and a C-terminal domain (CTD, also known as the regulatory or repressor domain (RD)), which are critical for RNA recognition. RIG-I and MDA5 also have two N-terminal CARD domains interacting with cascade signal molecules [89, 90]. LGP2 lacks an N-terminal CARD domain and is widely believed to regulate RIG-I and MDA5 [91]. In addition, RIG-I recognizes 5’-ppp-dsRNA, 5’-ppp-ssRNA, and blunt-ended short dsRNA (< 20 bp), while MDA-5 can recognize long dsRNA (> 1 kbp) with a blunt end, which is known to be generated by picornaviruses [91–93].

### The downstream pathways of RLRs signaling

**Fig. 3:**
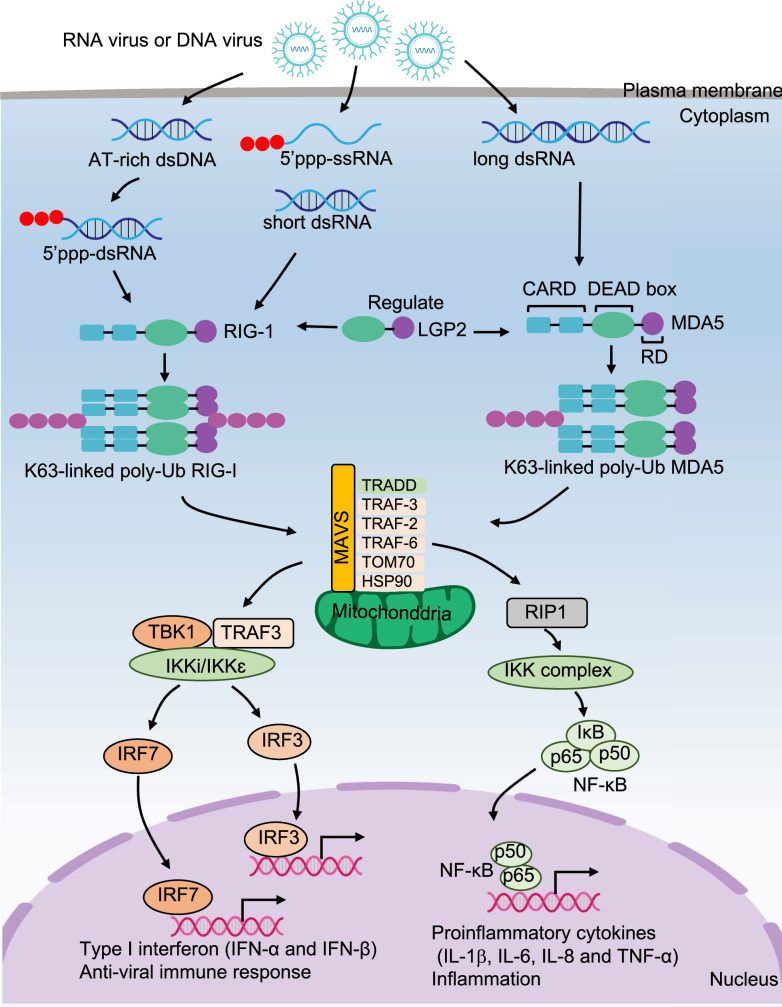
The signaling pathways for RLRs. After RNA virus or DNA virus infection, RIG-I recognizes 5’-ppp-dsRNA, 5’-ppp-ssRNA, and short dsRNA, while MDA-5 recognizes long dsRNA. Then RIG-I and MDA5 undergo lysine 63-linked polyubiquitin modification and form homotetramerization, respectively. Afterward, the homotetramerization recruits the MAVS and activates TRAF, which further activates TRAF3-TBK1-IKKε-IRF3/IRF7 pathway to produce type I IFNs, and activates RIP1-IKK complex-NF-κB pathway to induce proinflammatory cytokines secretion, including IL-1β, IL-6, IL-8 and TNF-α

RIG-I and MDA5 are activated by virus-derived RNA. They then undergo lysine 63-linked polyubiquitin modification and form homotetramerization. Afterward, homotetramerization recruits MAVS and activates TRAF, which further activates the TRAF3-TBK1-IKKε-IRF3/IRF7 pathway to produce type I IFNs and activates the RIP1-IKK complex-NF-κB pathway to induce proinflammatory cytokine secretion, including IL-1β, IL-6, IL-8 and TNF-α [92–94] (Fig. 3).

### Relationship between RLRs and autoimmune diseases

Aberrant RLR activation leads to several types of autoimmune diseases, such as amyopathic dermatomyositis (ADM) [95], type 1 diabetes [96], SLE [97], Aicardi-Goutieres syndrome (AGS) [98], and Singleton-Merten syndrome (SMS) [99]. An autoantibody to MDA5 was detected in a subpopulation of ADM patients, and the level of autoantibody was strongly correlated with disease severity [99, 100]. MDA5 was also reported to be associated with type 1 diabetes [88, 101, 102]. Several polymorphisms in *IFIH1*, which encodes MDA5, were found to be associated with resistance to type 1 diabetes, such as T946A, E627*, I923V, R843H, IVS8 + 1, and IVS14 + 1 [103–106]. Aberrant MDA5-mediated IFN production during picornavirus infection of pancreatic cells is one of the probable pathogeneses of the onset of type 1 diabetes [88]. In addition, other mutations of MDA5 were detected in patients with SLE, AGS, and SMS, and all these diseases exhibited a type I IFN signature [107–109]. In addition, RLRs are involved in adaptive immune responses. It has been reported that activated RLRs markedly attenuate TLR-induced Th1 and Th17 responses by suppressing IL-12b expression in DCs and macrophages. Meanwhile, activated RLRs also reshaped the immune system toward a Th2 response, possibly by inducing the polarizing cytokine IL-4 [110]. Furthermore, RIG-I activation promoted germinal center reactions and T follicular helper cell responses, resulting in the production of long-lasting antibodies and augmented antibody affinity [111]. However, aberrant activation of RLRs causing autoimmune diseases by regulating adaptive immune responses has not been reported. Hence, RLR-mediated excessive innate immune responses, inflammatory cytokines and autoantibody production are the main reasons for RLR-associated autoimmune diseases.

## CLRs and autoimmune diseases

### CLRs and their ligands

CLRs are characterized by containing at least one C-type lectin-like domain (CTLD) to bind carbohydrates, which is also named the carbohydrate recognition domain (CRD). CLRs are mainly expressed by myeloid cells and play important functions in the antifungal immune response [112]. CLRs can be divided into three subgroups based on their intracellular signaling motifs: CLRs with immunoreceptor tyrosine-based activation motif (ITAM)-like domains (also named hem-ITAM), for example, DC-associated C-type lectin-1 (Dectin-1); CLRs associated with ITAM containing the Fc receptor γ chain (FcR-γ), for example, Dectin-2, Dectin-3, and Mincle; and CLRs containing nonimmunoreceptor tyrosine-based motifs, for example, DC-specific ICAM3-grabbing nonintegrin (DC-SIGN) [113]. Dectin-1 recognizes β-glucans, while Dectin-2, Dectin-3, Mincle and DC-SIGN can recognize mannans and mannoproteins [113, 114].

### The downstream pathways of CLRs signaling

**Fig. 4:**
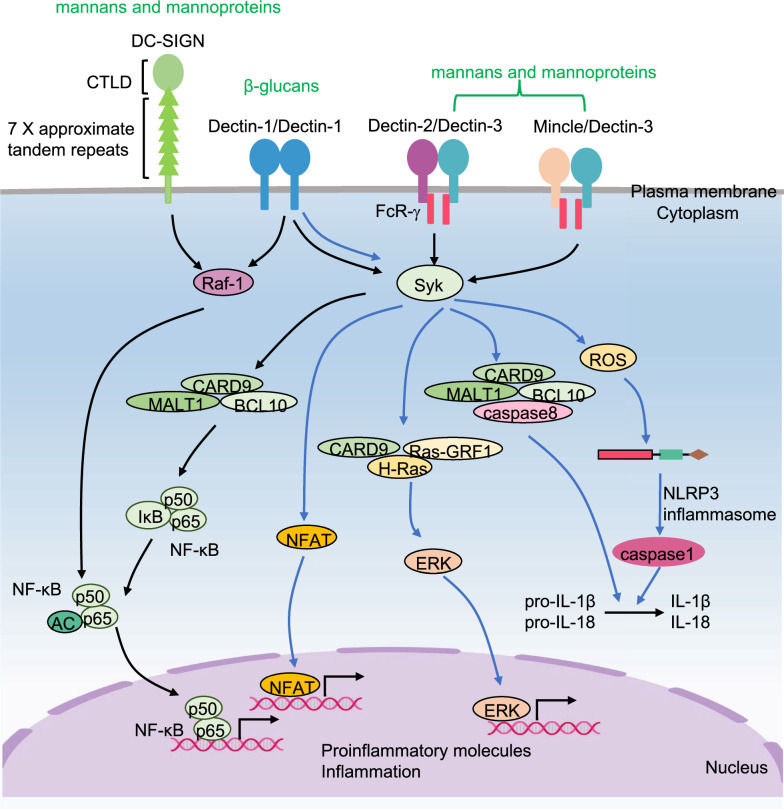
The signaling pathways for CLRs. Dectin-1, Dectin-2/Dectin3 and Mincle/Dectin-3 are activated by their ligands and then activate Syk. Afterward, Syk initiates caspase activation and recruits CARD9/BCL10/MALT1 complex to induce the expression of canonical NF-κB dependent proinflammatory molecules. Moreover, Dectin-1-Syk kinase signaling (i) initiates ROS production, which can mediate NLRP3 inflammasome formation and maturation of IL-1β and IL-18; (ii) promotes noncanonical caspase-8 inflammasome and then actives IL-1β production; (iii) initiates NFAT activation; (iv) mediates Ras-GRF1/H-Ras/CARD9 complex formation to induce ERK activation. In addition, Dectin-1 and DC-SIGN activate Ras, then induce Raf-1 mediated non-canonical NF-κB activation and subsequently promote proinflammatory molecule expression

Upon recognizing ligands, Dectin-1 homodimerizes and transduces intracellular signaling directly through hem-ITAM within its cytoplasmic tails, while Dectin-2 and Mincle heterodimerize with Dectin-3 and transduce intracellular signaling through FcR-γ containing ITAM [115]. CLR activation induces the tyrosine phosphorylation of hem-ITAM or ITAM by Src family kinases, leading to the recruitment and activation of Syk kinase. Subsequently, the kinase initiates caspase activation, recruits the complex of domain-containing protein 9 (CARD9)/B-cell lymphoma/leukemia 10 (BCL-10)/mucosa-associated lymphoid tissue 1 (MALT1), activates the canonical NF-κB signaling pathway and induces proinflammatory molecule production [116]. In addition, Dectin-1 activation also induces inflammatory cytokines by five other pathways: (i) Dectin-1-Syk kinase signaling initiates ROS production, which can mediate NLRP3 inflammasome formation and maturation of IL-1β and IL-18 [112]; (ii) Dectin-1-Syk kinase signaling is also involved in the formation of noncanonical caspase-8 inflammasome, which is responsible for active IL-1β production [117]; (iii) Dectin-1-Syk kinase signaling initiates nuclear factor of activated T cells (NFAT) activation in a calcineurin-dependent fashion, which integrates with NF-κB signaling [112]; (IV) Dectin-1 signaling pathway induces Syk-dependent activation of Ras-GRF1, which recruits H-Ras via CARD9 and ultimately leads to extracellular signal-regulated protein kinase (ERK) activation [112, 118]; (V) Dectin-1 transduces signals through the Raf-1-dependent pathway, as does DC-SIGN. Dectin-1 and DC-SIGN activate Ras, induce Raf-1-mediated noncanonical NF-κB signaling activation and subsequently promote proinflammatory molecule expression [119] (Fig. 4).

### Relationship between CLRs and autoimmune diseases

Excessive activation of CLRs can facilitate the development of autoimmune diseases by regulating innate and adaptive immunity [120–122]. Blockade of Dectin-1 can prevent SKG arthritis triggered by β-glucans by inhibiting the activation of synovial cells, including synovial macrophages/DCs and granulocytes [123]. Consistently, Dectin-1-deficient mice are resistant to both dextran sodium sulfate (DSS)- and CD45RB^high^ naïve CD4 T cell-induced colitis because of an increase in Treg cells [124]. Conversely, Dectin-1-activated DCs promote the Treg response to pancreatic β-cell antigen and prevent type 1 diabetes by producing large amounts of the immune regulatory cytokines IL-2, IL-10 and TGF-β1 [125]. These studies suggest that Dectin-1 plays different roles in different types of autoimmune diseases. Dectin-2 and Mincle activation were reported to induce EAU through the CARD9 signaling axis and IL-17 production [126, 127]. In addition, the expression of Dectin3 and Mincle on myeloid cells in the central nervous system is crucial for T cell recruitment and reactivation into a pathogenic Th17 phenotype, and their lower expression is associated with a drastic reduction in EAE incidence [128]. In addition, the activation of Mincle-Syk signaling aggravates intestinal inflammation by inducing macrophage pyroptosis and triggers Crohn's disease [129].

## Other sensors and autoimmune diseases

Nucleic acid sensing PRRs, including DNA sensors and RNA sensors, can trigger an immune response and are associated with autoimmune diseases. Apart from TLRs, RLRs and CLRs, there are several nucleic acid sensing PRRs, such as cyclic GMP-AMP (cGAMP) synthase (cGAS), stimulator of interferon genes (STING), AIM-like receptors (ALRs) and non-RLRs DExD/H-box family of helicases (DEAH-box helicases (DHX) family and DEAD-box helicases (DDX) family) [130].

### c-GAS-STING

cGAS, as a DNA sensor, recognizes cytoplasmic DNA of bacteria or viruses, catalyzes the synthesis of cGAMP, and then activates STING. STING recruits and activates TBK1, which in turn phosphorylates STING. Phosphorylated and activated STING recruits and licenses IRF3 for phosphorylation by TBK1. Finally, phosphorylated IRF3 enters the nucleus and induces IFN-β, CCL2 and CCL20 production [130]. The cGAS-STING pathway mediates protective immune defense against infection by a large variety of DNA-containing pathogens [131]. However, aberrant activation of the cGAS-STING pathway by self-DNA can also lead to autoimmune diseases, such as AGS [132]. Some molecules that can inhibit the cGAS-STING signaling pathway have been shown to alleviate autoimmune disease, as reviewed by Zhou et al. [133], supporting that cGAS-STING is one of the inducers of the development of autoimmune diseases.

### ALRs

ALRs consist of 4 proteins in humans, including gamma-interferon-inducible protein 16 (IFI16), absent in melanoma 2 (AIM2), myeloid cell nuclear differentiation antigen (MNDA) and interferon-inducible protein X (IFIX) [19]. After recognizing exogenous and endogenous DNA in the cytoplasm or nucleus, ALRs bind to ASCs and recruit pro-caspase-1 to form inflammasomes. Activated caspase-1 not only promotes the maturation of IL-1β and IL-18 but also promotes cell pyroptosis [130, 134]. In addition, some ALRs can also induce IFN-β production [134]. ALR activation is important for pathogen clearance under physiological conditions. However, aberrant ALR signaling can cause autoimmunity by producing excessive proinflammatory molecules and promoting autoreactive T and B cell responses [134, 135]. Until now, IFI16 and AIM2 have been reported to be involved in the pathogenesis of SLE and RA [134–137].

### Non-RLRs DExD/H-box family of helicases

The non-RLR DExD/H-box family of helicases, such as DHX9, DHX36, DDX41 and DDX3, has proinflammatory effects. DHX9 and DHX36 are known to interact with unmethylated CpG-DNA and then activate downstream NF-κB and IRF7 signaling [138, 139]. DDX41 recognizes ds-DNA or cGAMP and then interacts with STING and triggers TBK1 to activate downstream IRF3 signaling [138]. DDX3, as an RNA sensor, induces antiviral immunity in DCs [140]. However, the role of the non-RLR DExD/H-box family of helicases in the development of autoimmune diseases has not been reported and needs to be explored.

## Targeting innate immune sensors in the treatment of autoimmune diseases

Recently, an increasing number of inhibitors suppressing innate immune molecule responses have been studied, some of which have been applied to the treatment of autoimmune diseases. Here, we reviewed the research progress of these inhibitors according to their targets at three levels, namely, targeting receptors, signal transduction and terminal inflammatory molecules. The characteristics of inhibitors and their applications in different autoimmune diseases are summarized in Table 1.

**Table 1:** The characteristics and clinical applications of inhibitors targeting innate immune sensors in autoimmune diseases

Target	Inhibitor	Character	Application	Autoimmune disease	References
Inhibitors targeting innate receptors					
TLR4	NI-0101	Antibody	Phase II clinical trial	RA	[145]
TLR3/4	Baclofen	Small molecule	Phase III clinical trial	MS	[146, 147]
TLR7/9	IMO-3100	Oligonucleotides	Phase II clinical trial	Psoriasis	[209]
TLR7/9	Chloroquine	Small molecule	Clinical medicine	SLE and RA	[46, 141]
TLR7/9	Hydroxychloroquine	Small molecule	Clinical medicine	SLE and RA	[46, 141]
TLR7/9	Quinacrine	Small molecule	Clinical medicine	SLE and RA	[46, 141]
TLR7/8/9	CpG-52364	Small molecule	Phase I clinical trial	SLE	[143]
TLR7/8/9	IMO-8400	Oligonucleotides	Phase II clinical trial	Psoriasis	[144]
Inhibitors targeting signal transduction molecules					
IRAK4	PF-06650833	Small molecule	Phase II clinical trial	RA	[171]
IRAK4	BAY1834845	Small molecule	Phase I clinical trial	RA and psoriasis	[171]
Syk	R788	Small molecule	Phase I clinical trial	RA	[210]
Inhibitors targeting terminal proinflammatory cytokines					
TNF	Adalimumab	Monoclonal antibody	Clinical medicine	RA	[6, 202]
TNF	Certolizumab pegol	F(ab’) fragment of a humanized monoclonal antibody	Clinical medicine	RA	[6]
TNF	Etanercept	p75 (of TNFRII)-Fc (of IgG1) fusion protein	Clinical medicine	RA	[6, 202]
TNF	Golimumab	Monoclonal antibody	Clinical medicine	RA	[6]
TNF	Infliximab	Monoclonal antibody	Clinical medicine	RA	[6, 202]
IL-6R	Tocilizumab	Monoclonal antibody	Clinical medicine	RA	[197]
IL-6	Sarilumab	Monoclonal antibody	Phase III clinical trial	RA	[203]
IL-6	ALX-0061	Small molecule	Phase II clinical trial	RA	[203]
IL-6	Sirukumab	Monoclonal antibody	Phase II clinical trial	RA	[211]
IL-6	MEDI5117	Monoclonal antibody	Phase I clinical trial	RA	[212]
IL-6	Clazakizumab	Monoclonal antibody	Phase II clinical trial	RA	[213]
IL-6	Olokizumad	Monoclonal antibody	Phase II clinical trial	RA	[214]
IL-1	Anakinra	Recombinant	Clinical medicine	RA	[198]
IL-1	Rilonacept	Soluble decoy receptor	Clinical medicine	RA	[198]
IL-1	Canakinumab	Monoclonal antibody	Clinical medicine	RA	[198]
IFN-α	Sifalimumab	Monoclonal antibody	Phase II clinical trial	SLE	[215]
IFNAR	Anifrolumab	Monoclonal antibody	Phase III clinical trial	SLE	[216]
IFN-α	Rontalizumab	Monoclonal antibody	Phase II clinical trial	SLE	[204]
IL-18	Tadekinig alfa	Recombinant	Phase III clinical trial	NLRC4 and XIAP deficiency	[205]

## Inhibitors targeting innate receptors

### Inhibitors targeting TLRs

TLR antagonists, including small molecules, oligonucleotides, peptides, antibodies, proteins, nanoparticle inhibitors and drugs, have been developed for blocking ligand recognition or receptors dimerization, of which eight antagonists have been applied in the clinic or studied in clinical trials (Table 1). Three antimalarial drugs, chloroquine, hydroxychloroquine, and quinacrine, act as antagonists of TLR7, 8, 9 and have been used to treat SLE and RA. These drugs inhibit endosomal acidification and directly interact with nucleic acids to prevent their binding to endosomal TLRs [46, 141]. Clinical observations showed that these drugs could alleviate disease, prevent disease relapse, reduce disease complications and promote the prognosis of patients [142]. However, various adverse effects including gastrointestinal effects, myopathy, cardiotoxic effects, and retinopathy limit their clinical applications [142]. CpG-52364 (a quinacrine derivative) was more effective and safer compared to hydroxychloroquine in an animal study, and its Phase I clinical trial in SLE patients was completed [143]. Two TLR antagonists have finished phase II clinical trials in psoriasis patients. The results showed that the Psoriasis Area Severity Index score was decreased in psoriasis patients treated with immune modulatory oligonucleotide- (IMO-) 3100, an antagonist of TLR7 and TLR9 [141]. IMO-8400, an oligonucleotide-based antagonist of TLR7,8,9, was reported to be safe and effective in the treatment of plaque psoriasis patients, only with mild adverse effects [144]. Moreover, several clinical trials to evaluate the effects of NI-0101 (a humanized monoclonal antibody that interferes with TLR4 dimerization) and baclofen (a TLR3 and TLR4 signaling small molecule inhibitor) are in the progress in RA patients and MS patients [145–147].

In addition, some TLR inhibitors have been verified to be effective in the treatment of autoimmune diseases in mouse models. For example, ( +)-naltrexone [( +)-NTX], an antagonist of TLR2 and TLR4, was effective at treating MS-related memory deficits with both lower- and higher-dose in EAE mice but failed to alleviate EAE-induced motor deficits [148]. TLR7/9 antagonists, compound 29 and IRS-954 (immunoregulatory DNA sequence-954), showed therapeutic efficacy in a preclinical murine model of psoriasis and lupus, respectively [149, 150]. Wang et al. found that total coumarins from Urtica dentata Hand could prevent murine autoimmune diabetes by suppressing the TLR4 signaling pathways in DCs [151]. In addition, drug-coated nanoparticles tend to accumulate in inflammatory joints and thus possess augmented effectiveness. Opuntiol-coated silver and gold nanoparticles (OP-AgNPs and OP-AuNPs) were reported to prevent disease progression in complete Freund's adjuvant (CFA)-induced arthritic rats by suppressing the expression of TLR2 and TLR4 [152]. Therefore, PRR inhibitors coated nanoparticles with increasing bioavailability, stability and targeted drug delivery capacity are promising strategies for autoimmune diseases.

### Inhibitors targeting NLRs

Overactivation of the NLRP3-containing inflammasome has been widely associated with various autoimmune diseases, including SLE, RA, SSc, IBD, MS and autoimmune thyroiditis (AIT) [153–159]. Thus, targeting NLRP3 is a promising strategy to treat autoimmune diseases. Indeed, Tofacitinib, a Janus kinase (JAK) inhibitor, was recently reported to alleviate collagen-induced arthritis (CIA) by inhibiting the NLRP3 inflammasome [160]. RRx-001, a well-tolerated anticancer drug undergoing phase III clinical trials, was reported to specifically inhibit the activation of the NLRP3 inflammasome and attenuate the symptoms of dextran sulfate sodium (DSS)-induced colitis and EAE in mice [161]. CY-09, which can inhibit NLRP3 ATPase activity, was shown to have remarkable therapeutic effects in mouse models of CAPS and type 2 diabetes [162]. In addition, OLT1177, thiolutin and MCC950 significantly ameliorated the clinical signs of EAE mice by specifically targeting NLRP3 inflammasome [160, 161, 163]. Although a large number of mouse studies have shown that NLRP3 inhibitors are effective in the treatment of autoimmune diseases, the clinical use of NLRP3 inhibitors has not been reported.

### Inhibitors targeting RLRs

MDA5 is involved in several autoimmune diseases, including type 1 diabetes, MS, psoriasis and SLE [96, 164–166]. There is no report of inhibitors targeting MDA5. However, some MDA5-binding proteins could be exploited as therapeutic targets by blocking their interactions. For example, ARL5B was found to prevent the interaction between MDA5 and dsRNA by binding to MDA5 [167]. DNAJB1 disrupted MDA5 multimer formation after binding to MDA5, resulting in the suppression of type I IFN production [96]. In addition, ubiquitin-specific protease 3 (USP3), a deubiquitinase, was reported to bind to MDA5, remove K63-polyubiquitin chains on the N-terminal CARDs, and then inhibit MDA5 activity [168]. The treatment effect of small molecules targeting MDA5 binding proteins needs further study.

### Inhibitors targeting CLRs

Dysregulation of CLRs is associated with the development of autoimmune diseases, allergies, and cancer [121]. Although several agonists of CLRs for cancer treatment have been applied in the clinic [169], targeting CLRs in the treatment of autoimmune diseases are still in the early experimental stage. For example, laminarin, a Dectin-1 antagonist, was reported to suppress the development of DSS-induced colitis by inducing Treg cells in mice [124].

## Inhibitors targeting signal transduction molecules

### Inhibitors targeting TLR signal transduction molecules

Several molecules, including MyD88, IRAK4, TRAF6, TAK1, TRIF, TBK1 and NF-κB, are involved in TLR-associated signal transduction. Inhibitors targeting these molecules have been explored in the treatment of autoimmune diseases. However, only three IRAK4 inhibitors have entered clinical trials. The results of a 12-week phase II clinical trial showed that activated RA patients treated with once daily doses of PF-06650833 (Pfizer) exhibited reduced disease activity scores and an improved rate of ACR50 responses compared to the placebo control group. Only 6.4% (12/187) of participants dropped off the study due to adverse effects [170]. BAY1834845 (Bayer) and BAY1830839 (Bayer) have completed phase I clinical trials in healthy volunteers, and phase I/II clinical trials are under way [171].

Most inhibitors are at the interface between basic and clinical research, using mouse models to explore their treatment efficiency. For example, the MyD88 function inhibitors, c(MyD4–4) and TJ-M2010–6, were reported to alleviate the severity of EAE and reduce the onset of autoimmune diabetes in experimental NOD mice, respectively [172, 173]. IRAK4 inhibitors, HS-243, ND-2158 and ND-2110, were shown to suppress LPS-induced proinflammatory cytokines and relieve the symptoms in rheumatoid arthritis fibroblast-like synoviocytes (RAFLSs) and CIA mouse models [174–176]. Two NF-κB inhibitors, polyphyllin and taraxasterol, could block the production of proinflammatory effectors obviously in CIA mice [177, 178]. Furthermore, TAK1 inhibitors, takinib and oxozeaenol, were reported to alleviate the severity of CIA in mouse model [179] and reduce the onset of autoimmune diabetes in NOD mice [180]. Remarkably, siRNAs, which serve as treatments for autoimmune disease, were applied in mouse models. Adenoviral-mediated siRNA against TAK1 treatment helped to control joint inflammation by suppressing JNK activation and the expression of proinflammatory cytokines in CIA mice [181]. Another study performed by Wang et al. showed that siRNAs targeting the *TRIF* gene alleviated the severity of EAE in a mouse model by reducing IFN-γ and IL-2 levels [182]. Therefore, further studies are required to accelerate the clinical application of inhibitors targeting TLR signal transduction molecules.

### Inhibitors targeting NLR signal transduction molecules

RIPK2, which mediate proinflammatory signaling from NOD1 and NOD2, is an emerging therapeutic target in autoimmune diseases [183–185]. A selective RIPK2 kinase inhibitor, WEHI-345, was reported to delay RIPK2 ubiquitylation, prevent cytokine production and ameliorate EAE in mice [186]. Remarkably, four RIPK2 inhibitors (ponatinib, regorafenib, gefitinib and erlotinib), which are approved by the US Food and Drug Administration, are used clinically against various forms of cancer [183, 187, 188]. Considering the anti-inflammatory effect of these drugs by inhibiting NLR signal transduction, their therapeutic potential in autoimmune diseases is worth further investigation.

### Inhibitors targeting RLR and CLR signal transduction molecules

Reports related to inhibitors targeting RLR signal transduction molecules are rare. E3 ubiquitin ligases, RNF125, A20, MARCH5, SMURF2, and AIP4, are implicated in inhibiting RLR signaling by targeting MAVS [189], which could be exploited as therapeutic targets for the treatment of autoimmune disease. Syk kinase is a key signal transduction molecule of the CLR signaling pathway. It has been reported that R788, a selective Syk inhibitor, obviously delays spontaneous diabetes onset in NOD mice [190] and prevents and suppresses skin injury in lupus MRL/lpr mice [191]. Furthermore, R788 was reported to be effective in RA patients in a randomized clinical phase II trial [192], but unfortunately, the clinical use of R788 was hampered due to obvious side effects [193].

### Inhibitors targeting terminal proinflammatory cytokines

Most innate immune molecules function by producing proinflammatory cytokines. Thus, inhibiting terminal proinflammatory cytokines is considered an effective and rapid treatment strategy for autoimmune diseases. To date, nine proinflammatory cytokine inhibitors targeting TNF, IL-6R and IL-1 have been used in the clinical treatment of RA [194–200] (Table 1). Six IL-6 blockers for SLE treatments and one IL-18 inhibitor for patients with NLRC4 and XIAP deficiency (monogenic IL-18 associated autoinflammatory conditions) are in phase II/III clinical trials (Table 1) [6, 198, 201–205]. Although the administration of the proinflammatory cytokine blockade increased therapeutic efficacy on autoimmune diseases, the serious side effects, for example, increased risk of infection, malignancy and induction of multiple sclerosis, lupus, psoriasis, or heart failure, and the expensive costs limited their clinical applications [206–208]. Therefore, it is of great significance to explore safer, more effective and more economical proinflammatory cytokines blockade strategies for autoimmune diseases.

## Conclusions

PRRs are capable of activating the inflammatory pathway in innate immune cells and collaborating with antigen receptors to enhance activation in adaptive immune cells. Both synergistically and individually contribute to the initiation and/or development of autoimmune diseases. So far, a total of 30 inhibitors that inhibit innate immune molecular responses are in clinical use or undergoing clinical trials, several of which have the obvious advantages of better clinical efficacy. However, the serious side effects and the expensive costs are still considerable problems to be solved. In addition, many small molecule inhibitors which work well in animal models of autoimmune diseases are ideal candidates for further study. Therefore, PRRs might be considered potent targets for manipulating the kinetic status of the autoimmune response. In the future, it is worth exploring more molecules and related pathways that are critical for both innate and adaptive immunity. It should also evaluate the efficacy of PRR inhibitors combined with conventional drugs for anti-autoimmune diseases.

## Data Availability

Not applicable.

## Abbreviations

PRRs: Pattern recognition receptors
TLRs: Toll-like receptors
NLRs: Nod-like receptors
RLRs: RIG-I like receptors
CLRs: C-type lectin receptors
APCs: Antigen-presenting cells
PAMPs: Pathogen-associated molecular patterns
DAMPs: Danger-associated molecular patterns
SLE: Systemic lupus erythematosus
RA: Rheumatoid arthritis
LRR: Leucine-rich repeats
TIR: Toll interleukin-1 receptor homology
DCs: Dendritic cells
NK: Natural killer
LPS: Lipopolysaccharide
dsRNA: Double-stranded RNA
ssRNA: Single-stranded RNA
MyD88: Myeloid differentiation primary response 88
Mal: MyD88 adapter like
TRIF: TIR-domain containing adapter inducing interferon-β
TRAM: TRIF-related adaptor molecule
IRAK-4: Interleukin 1 receptor associated kinase 4
TRAF6: TNF receptor associated factor 6
TAK1: TGF-β-activated kinase 1
TAB: TAK1-binding proteins
RIP-1: Receptor-interacting protein-1
TBK1: TANK-binding kinase 1
AIH: Autoimmune hepatitis
SSc: Systemic sclerosis
EAU: Experimental autoimmune uveitis
EAE: Experimental autoimmune encephalomyelitis
MS: Multiple sclerosis
FA: Food allergy
NOD: Nucleotide-binding oligomerization domain
AD: Acidic transactivation domain
BIR: Baculovirus inhibitor of apoptosis protein repeat domain
CARD: Caspase activation and recruitment domain
PYD: Pyrin domain
MTS: Mitochondria-localization sequence
CIITA: MHC-II transcription activator
NAIP: Neuronal apoptosis inhibitory protein
MDP: Muramyl dipeptide peptidoglycan
ROS: Reactive oxygen species
RIPK2: Receptor-interacting serine/threonine-protein kinase 2
MAVS: Mitochondrial antiviral signaling
ASCs: Apoptosis-associated speck-like protein
UC: Ulcerative colitis
CD: Crohn’s disease
FCAS: Familial cold autoinflammatory syndrome
RIG-I: Retinoic acid-inducible gene I
MDA5: Melanoma differentiation-associated gene 5
LGP2: Laboratory of genetics and physiology 2
CTD: C-terminal domain
RD: Repressor domain
AGS: Aicardi-Goutieres syndrome
SMS: Singleton-Merten syndrome
CTLD: C-type lectin-like domain
CRD: Carbohydrate recognition domain
ITAM: Immunoreceptor tyrosine-based activation motif
Dectin-1: DC-associated C-type lectin-1
FcR-γ: Fc receptor γ chain
DC-SIGN: DC-specific ICAM3-grabbing nonintegrin
CARD9: Caspase activation, recruits the complex of domain-containing protein 9
BCL-10: B-cell lymphoma/leukemia 10
MALT1: Mucosa-associated lymphoid tissue 1
NFAT: Nuclear factor of activated T cells
ERK: Extracellular signal-regulated protein kinase
cGAMP: Cyclic GMP-AMP
cGAS: Cyclic GMP-AMP synthase
STING: Stimulator of interferon genes
ALRs: AIM-like receptors
IFI16: Gamma-interferon-inducible protein 16
AIM2: Absent in melanoma 2
MNDA: Myeloid cell nuclear differentiation antigen
IFIX: Interferon-inducible protein X
IMO: Immune modulatory oligonucleotide
IRS-954: Immunoregulatory DNA sequence-954
CFA: Complete Freund's adjuvant
AIT: Autoimmune thyroiditis
JAK: Janus kinase
CIA: Collagen-induced arthritis
DSS: Dextran sulfate sodium
USP3: Ubiquitin-specific protease 3
